# Reliability of maximal mitochondrial oxidative phosphorylation in permeabilized fibers from the *vastus lateralis* employing high‐resolution respirometry

**DOI:** 10.14814/phy2.13611

**Published:** 2018-02-21

**Authors:** Daniele A. Cardinale, Kasper D. Gejl, Niels Ørtenblad, Bjorn Ekblom, Eva Blomstrand, Filip J. Larsen

**Affiliations:** ^1^ Åstrand Laboratory Department of Sport and Health Sciences The Swedish School of Sport and Health Sciences Stockholm Sweden; ^2^ Elite Performance Centre Bosön ‐ Swedish Sports Confederation Lidingö Sweden; ^3^ Department of Sports Science and Clinical Biomechanics University of Southern Denmark Odense Denmark

**Keywords:** Mitochondria, reliability, standard error of the measurement

## Abstract

The purpose was to assess the impact of various factors on methodological errors associated with measurement of maximal oxidative phosphorylation (OXPHOS) in human skeletal muscle determined by high‐resolution respirometry in saponin‐permeabilized fibers. Biopsies were collected from 25 men to assess differences in OXPHOS between two muscle bundles and to assess the correlation between OXPHOS and the wet weight of the muscle bundle. Biopsies from left and right thighs of another five subjects were collected on two occasions to compare limbs and time‐points. A single muscle specimen was used to assess effects of the anesthetic carbocaine and the influence of technician. The difference in OXPHOS between two fiber‐bundles from the same biopsy exhibited a standard error of measurement (SEM) of 10.5 pmol · s^−1^ · mg^−1^ and a coefficient of variation (CV) of 15.2%. The differences between left and right thighs and between two different time‐points had SEMs of 9.4 and 15.2 pmol · s^−1^ · mg^−1^ and CVs of 23.9% and 33.1%, respectively. The average (±SD) values obtained by two technicians monitoring different bundles of fibers from the same biopsy were 31.3 ± 7.1 and 26.3 ± 8.1 pmol · s^−1^ · mg^−1^. The time that elapsed after collection of the biopsy (up to a least 5 h in preservation medium), wet weight of the bundle (from 0.5 to 4.5 mg) and presence of an anesthetic did not influence OXPHOS. The major source of variation in OXPHOS measurements is the sample preparation. The thigh involved, time‐point of collection, size of fiber bundles, and time that elapsed after biopsy had minor or no effect.

## Introduction

Mitochondrial coupling of substrate oxidation to phosphorylation of ADP in order to produce free ATP requires pumping of protons over the inner mitochondrial membrane and transfer of electrons through the electron transport chain to oxygen as the final acceptor. Thus, assuming that all oxygen consumed is due to this process and that the mitochondria are fully coupled, maximal oxygen consumption is a reliable indicator of mitochondrial ATP production (Wibom and Hultman 1990). Skeletal muscle mitochondria are highly responsive to various stimuli, including drugs, nutritional status, exercise, and certain diseases and it is crucial that changes measured in their oxidative phosphorylation in situ mirror functional and morphological alterations in vivo.

High‐resolution respirometry is commonly used to assess mitochondrial respiration, either by the isolated mitochondria (Tonkonogi et al. 1997) or in saponin‐permeabilized muscle fibers (Kuznetsov et al. 2008; Pesta and Gnaiger 2012). Using different combinations of specific substrates and inhibitors, the oxidative phosphorylation of specific complexes of the electron transfer system can be assessed mimicking mitochondrial physiology, for example, in exercising skeletal muscle. The approach using saponin‐permeabilized bundle of fibers requires less muscle and allows in situ assessment of maximal oxidative phosphorylation (OXPHOS) with preservation of the mitochondrial reticulum. However, mitochondrial ATP production (Wibom and Hultman 1990), apparent oxygen (O_2_) affinity (p50_mito_) (Gnaiger et al. 1998) and the P/O ratio can only be determined employing isolated mitochondria. Although both valid, these two techniques are not interchangeable, possessing their individual advantages and disadvantages (Picard et al. 2010, 2011).

The importance of OXPHOS measurements in health and disease make it crucial that alterations in this parameter actually mirror functional and morphological changes. However, little is known about the reliability of OXPHOS measurement, using fiber bundles. For example, even though the distribution of fiber types in the *vastus lateralis* muscle of the right and left human legs is similar (Blomstrand and Ekblom 1982), there might still be differences in OXPHOS. This is a particularly important consideration when the one leg is exercised and the contralateral leg of the same subject is used as the control (Andersen et al. 1985). Thus, specific measures of reliability associated with repeated determination of OXPHOS, comparison of the right and left legs, which is assumed equal, variability with measurements at different time‐points and over time, as well as influence of the wet weight of the fiber bundle on determined OXPHOS, is needed.

For these reasons, here we compared OXPHOS values obtained with biopsies from human *vastus lateralis* muscle in four different ways, that is, the values for two bundles of fibers in the same biopsy; the values for the left and right thighs of the same subject; the values obtained at two time‐points 27 ± 6 days apart; as well as the measurements by two different technicians. In addition, the potential effects of sample weight, time that elapsed after collection of the biopsy, and the use of anesthetic were evaluated.

## Material and Methods

### Study design and the characteristics of our subjects

Single muscle biopsies collected from the right *vastus lateralis* of 25 well‐trained young men (age 24.7 ± 4.5 years, body mass 75.5 ± 6.3 kg, height 183 ± 6.0 cm) were utilized to assess differences in OXPHOS between two muscle bundles from the same specimen, as well as to assess the correlation between OXPHOS and the wet weight of the muscle bundle. Biopsies were also collected from the left and right thighs of another five healthy recreationally active subjects (four men and one women; age 25.6 ± 2.4 years, body mass 78.5 ± 16.2 kg, height 180.9 ± 11.8 cm) on two occasions 27 ± 6 days apart in order to compare both limbs and time‐points. These subjects were instructed not to change their physical activity pattern between these two laboratory visits. Furthermore, a single muscle specimen from an elderly individual (age 75 years, body mass 82 kg, height 176 cm) was analyzed repeatedly by two technicians to assess the impact of differences in sample preparation (including dissection, weighing and permeabilization), as well as any potential direct effect of the anesthetic carbocaine on OXPHOS.

A screening survey indicated that all of these volunteers were healthy. They were informed of the possible risks and discomfort involved prior to providing their written consent to participate. This study was preapproved by the Swedish Regional Ethics Committee (Project‐ID 2015/644‐31) and the Ethics Committee of Southern Denmark (Project‐ID S‐20150034) and adhered to the principles formulated in the Declaration of Helsinki.

### Collection of muscle biopsies

Muscle biopsies were collected as described by Ekblom (2017). In brief, following application of local anesthesia (2–4 mL carbocaine, 20 mg/mL; AstraZenenca, Södertälje, Sweden) to the middle portion of the *vastus lateralis* (about one‐third of the distance from the upper margin of the patella to the anterior superior iliac spine)*,* an incision ~ 1.0 cm was made through the skin and fascia and a muscle biopsy (50–100 mg) taken at a depth of 2–3 cm with a Weil–Blakesley chonchotome (Henriksson 1979) or a Bergström needle (Bergström 1962). The muscle samples were placed rapidly into ice‐cold BIOPS solution (10 mmol/L Ca^2+^/EGTA buffer, pH 7.1, 20 mmol/L imidazole, 50 mmol/L K^+^‐4‐morpholinoethanesulfonic acid (Mes), 0.5 mmol/L dithiothreitol, 6.56 mmol/L MgCl_2_, 5.77 mmol/L ATP, and 15 mmol/L phosphocreatine), in which they were maintained constantly thereafter, until permeabilization was initiated.

### Saponin‐permeabilization

A portion of the muscle biopsy (~ 5–10 mg wet weight) was transferred onto a small, ice‐cold petri dish and thereafter disrupted with a forceps and needles. The longest bundles of muscle fibers that appeared intact under the light microscope were incubated with gentle shaking for 20 min in ice‐cold BIOPS solution containing 10 *μ*L of 5 mg · mL^−1^ saponin per mL. Subsequently, these fibers were washed for 10 min in ice‐cold mitochondrial respiration medium (MiR05; 0.5 mmol/L EGTA, 3 mmol/L MgCl_2_, 60 mmol/L K‐lactobionate, 20 mmol/L taurine, 10 mmol/L KH_2_PO_4_, 20 mmol/L HEPES, 110 mmol/L sucrose and 1 g · L^−1^ essentially fatty acid‐free bovine serum albumin, adjusted to pH 7.1), blotted for ~ 5 sec on filter paper, and finally weighed before being transferred in the MiR05 medium into the chambers of a high‐resolution respirometer.

### Mitochondrial respiration

Mitochondrial respiration was monitored in a high‐resolution respirometer (Oroboros Oxygraph, Paar, Graz, Austria) at 37°C, magnetic stirring at 750 rpm, and two chambers (channel A and channel B) allowing assessment of two samples at the same time, with data collection once every second. Oxygen consumption and zero‐drift of the oxygen electrode were calculated using DatLab 5.2 software (Oroboros). In a separate experiment, five different known oxygen tensions (from 250 to ~ 0 nmol · mL^−1^) were used to calibrate the diffusion of oxygen into the chamber. In the presence of complete saturation with ADP (2.5 mmol/L), OXPHOS was obtained by titration with octanoylcarnitine (0.2 mmol/L), pyruvate (5 mmol/L), malate (2 mmol/L), glutamate (10 mmol/L), and succinate (10 mmol/L). Glutamate was not titrated when comparing left and right (R) tight and between time‐points. In addition, titrating a dose of carbocaine (81 mmol/L) which was comparable to the one used prior to biopsy collection, the effect of anesthetic on OXPHOS was assessed. In general, the mitochondria in our permeabilized fiber bundles showed an intact outer mitochondrial membrane as evident by the modest increase (mean 1.1 ± 8.2%, *P *= 0.67) in respiratory rate when exogenous cytochrome c was added during the respiratory experiments. Mitochondrial coupling was also well preserved and respiratory control ratios (OXPHOS divided by LEAK‐respiration) was 8.0 ± 4.1. Overall, this indicates that the mitochondria in our preparations were intact and of good quality.

### Statistical analyses

Assessment of skewness with Levene's test revealed that all the data exhibited equal variance. The presence of outliers in the data set was tested with the ROUT method and Grubbs’ method, using Prism v. 6.07 (GraphPad Software Inc., La Jolla, CA). Differences in OXPHOS between two muscle bundles from the same biopsy were compared using the paired *t*‐test. To assess differences between the left and right thighs and between the two time‐points, a two‐way repeated measures analysis of variance (ANOVA) was employed. To follow up any significant interactions observed, simple effect tests were applied. The differences in OXPHOS between the two technicians and in the presence and absence of carbocaine were examined with Student's paired *t*‐test. A two‐tailed *P* value < 0.05 was considered significant.

The methodological error in OXPHOS measurement was quantified by calculating the standard error of measurement (SEM) according to equation (1) (Weir 2005):

(1) $$SEM=\sqrt{\frac{\sum d^{2}}{2n}}$$

The coefficient of variation (CV) for repeated measurements was calculated as the ratio between this SEM and the mean value, expressed as a percentage. Sample size required to compare two populations means *μ*0 and *μ*1, with two‐sided alternatives with Type I error 0.05 (*α*) and power 0.80 (*β*) was calculated according to van Belle and Millard (1998):

(2) $$n=\frac{2{\left(\frac{z_{1}-\alpha }{2}+z1-\beta \right)}^{2}}{\left(\frac{\mu 0-\mu 1}{\mathrm{SEM}}\right)}$$

All statistical analyses were carried out, using the SPSS software, v.21 (SPSS Inc., Chicago, IL) and Prism v. 6.07 (GraphPad Software Inc.).

## Results

### Repeated determination of OXPHOS is associated with a relatively large variability between fiber bundles

**Figure 1:**
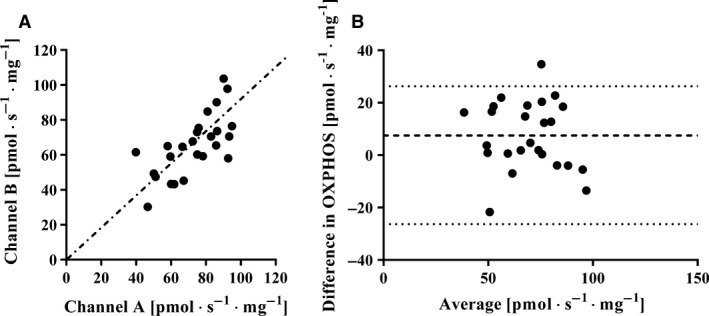
Left panel: Maximal mitochondrial oxidative phosphorylation (OXPHOS) (pmol · s^−1^ · mg^−1)^ measured simultaneously on two different fibers bundles in two different channels (A and B) of a high‐resolution respirometer (*n* = 25). Each filled circle represents one individual and the dotted line shows the line of identity. Right panel: Bland–Altman plot showing the spread (channel A–B) from the average bias and the limit of agreement (2 · the standard deviation of the bias).

To test the OXPHOS reliability in repeated measurements, OXPHOS was determined in two fiber bundles from the same biopsy, using a total of 25 subjects. OXPHOS in these biopsies averaged 69.2 ± 17.0 pmol · s^−1^ · mg^−1^. The mean and standard deviation (SD) for the difference between the two bundles was 2.9 ± 14.7 pmol · s^−1^ · mg^−1^ (*P *> 0.05), the SEM 10.5 pmol · s^−1^ · mg^−1^ and CV 15.2% (Table 1). The individual values are shown in Figure 1.

**Table 1:** Summary of all the standard errors of measurement and coefficient of variation

Description	*n*	Sample 1 mean ± SD [pmol · s^−1^ · mg^−1^]	Sample 2 mean ± SD [pmol · s^−1^ · mg^−1^]	SEM [pmol · s^−1^ · mg^−1^]	CV [%]
Two preparations from the same biopsy sample	25	72.9 ± 16.1	65.4 ± 17.4	10.5	15.2
Two preparations, one from left and one from right thigh	5	42.0 ± 8.9	36.3 ± 5.4	9.4	23.9
Two preparations from two time‐points	5	42.0 ± 8.9	49.8 ± 16.7	15.2	33.1

*n*: number of subjects; SD; standard deviation; SEM: standard error of the measurement; CV: coefficient of variation.

### Similar OXPHOS measurement is obtained when comparing right and left legs

**Figure 2:**
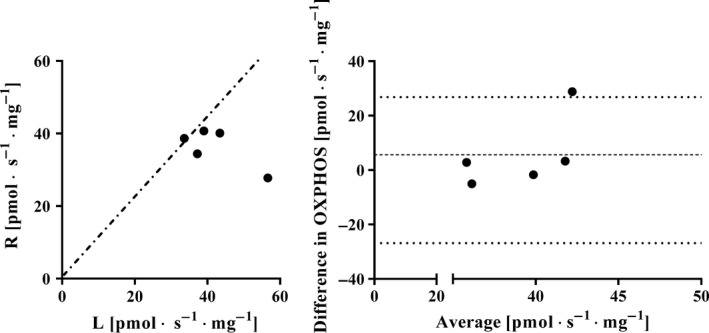
Left panel: Maximal mitochondrial oxidative phosphorylation (OXPHOS) (pmol · s^−1^ · mg^−1^) by bundles of fibers collected from the left (L) and right (R) *vastus lateralis* muscles from the same subject. Each filled circle represents one individual and the dotted line shows the line of identity. Right panel: Bland–Altman plot showing the spread (L‐R) from the average bias and the limit of agreement (2 · the standard deviation of the bias).

Additionally, to assess if OXPHOS differs between limbs OXPHOS from specimens collected from the left and right *vastus lateralis* muscle of the same subject was compared. The average OXPHOS values (± SD) for the left and right thighs were 42.0 ± 8.91 and 36.3 ± 5.38 pmol · s^−1^ · mg^−1^, respectively (*P *> 0.05); SEM = 9.4 pmol · s^−1^ · mg^−1^, CV = 23.9% (Table 1). The individual values are presented in Figure 2.

### Determination of OXPHOS is similar at two different time‐points

**Figure 3:**
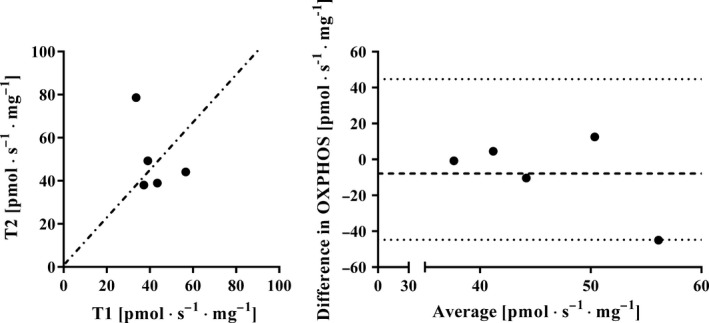
Left panel: Maximal mitochondrial oxidative phosphorylation (OXPHOS) (pmol · sec^−1^ · mg^−1^) by fibers collected from the same muscle 27 ± 6 days apart (*n* = 5). Each filled circle represents one individual and the dotted line shows the line of identity. Right panel: Bland–Altman plot showing the spread (T1‐T2) from the average bias and the limit of agreement (2 · the standard deviation of the bias).

To assess the variance in OXPHOS in two biopsies obtained at different time‐points, OXPHOS was assessed in two biopsies obtained with several weeks in between. The average (± SD) OXPHOS values for biopsies collected from the left thigh with 27 ± 6 days in between were 42.0 ± 8.9 and 49.8 ± 16.7 pmol · s^−1^ · mg^−1^, respectively. Analysis by ANOVA showed no main effect of time (*P *> 0.05) or interaction between time and leg (*P *> 0.05). The SEM was 15.2 pmol · s^−1^ · mg^−1^ with a CV of 33.1% (Table 1). Similar values were found for repeated biopsies from the right thigh, SEM was 14.9 pmol · s^−1^ · mg^−1^ with a CV of 31.9% (data not shown). The individual values for the left thigh are documented in Figure 3.

### Technician sample preparation technique can be a source of variation in OXPHOS measurement

**Figure 4:**
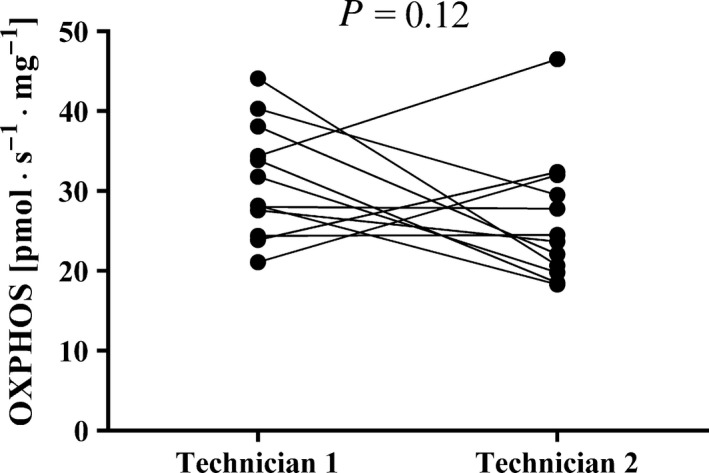
The variance in maximal mitochondrial oxidative phosphorylation (OXPHOS) by bundles of fibers from the same biopsy as measured by two experienced technicians. Each measurement is represented by a filled circle. Two measurements performed in the same respirometer at the same time of the day, but with permeabilization by the different technicians are connected with a line. *P* indicates the level of significance (no significant difference was observed).

To evaluate whether the OXPHOS assessment is influenced by the technician work, two experienced technicians dissected out, permeabilized, and weighed different fiber bundles (12 for each technician) from the same biopsy. The average (± SD) values obtained from technician 1 was 31.3 ± 7.1 pmol · s^−1^ · mg^−1^ which and 26.3 ± 8.1 pmol · s^−1^ · mg^−1^ from technician 2 with a tendency for difference between technicians (*P *= 0.12). The individual values can be seen in Figure 4.

### The wet weight of the fiber bundle has no effect on the determination of OXPHOS

**Figure 5:**
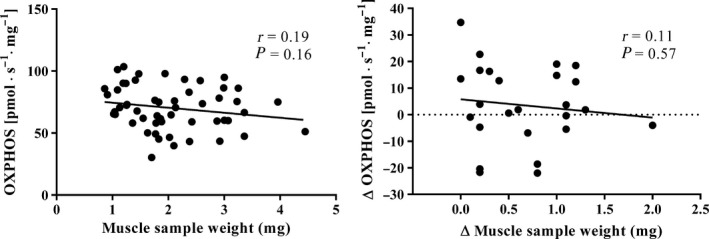
The left panel depicts the correlation between maximal mitochondrial oxidative phosphorylation (OXPHOS) and the wet weight of the fiber bundle in 50 permeabilized samples. Each measurement is represented by a filled circle and the black continuous line shows the trend, with the Pearson product moment correlation coefficient (*r*) also being indicated. The right panel depicts the difference in OXPHOS (*Y* axis) between two fiber bundles of different wet weight (*X* axis) from the same subject (*n* = 25). Each measurement is represented by a filled circle and the black continuous line depicts the trend, with the Pearson product moment correlation coefficient (*r*) also being indicated.

In bundles containing hundreds of fibers, limited availability of O_2_ to mitochondria located at some distance from the pore may reduce their OXPHOS capacity. However, we found no significant correlation between the wet weight of the fiber bundle and OXPHOS (Fig. 5, left panel) or between the difference in OXPHOS and difference in fiber bundle wet weight in two fiber bundles for the same subject (Fig. 5, right panel).

### OXPHOS remains unchanged for at least 5 h after collection of the biopsy

**Figure 6:**
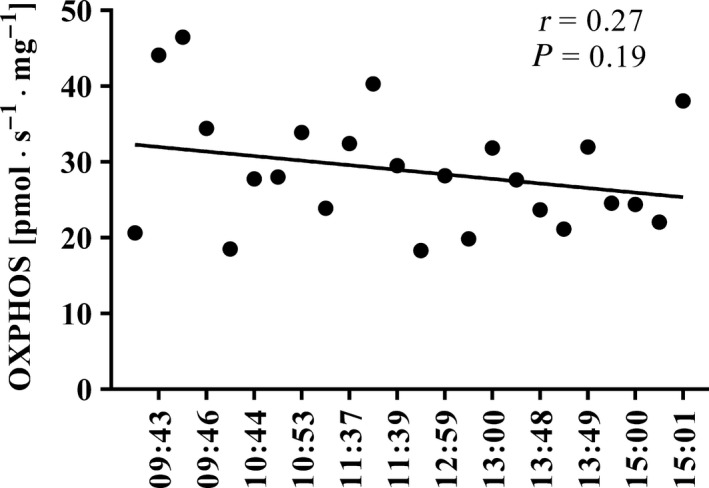
Values obtained upon 24 repeated measurements of maximal mitochondrial oxidative phosphorylation (OXPHOS) by bundles of fibers from the same sample at various times after collection of the biopsy. Each measurement is represented by a filled circle and the black continuous line depicts the trend, with the Pearson product moment correlation coefficient (*r*) also being indicated.

With a fresh muscle sample kept in ice‐cold BIOPS medium specifically designed to preserve mitochondrial function (see Material and methods) OXPHOS remained the same for at least 5 h (Fig. 6). It should be emphasized that the fiber bundle was permeabilized immediately prior to the starts of the measurement.

### The anesthetic carbocaine does not affect determination of OXPHOS

It is not known whether local anesthetics alters mitochondrial physiology and changes OXPHOS. To study this, carbocaine in a dose that resembled the amount used during local anesthesia was injected directly into the respiration chamber to examine its potential effects on OXPHOS. The average (± SD) of twenty measurements with and without this addition were 27.3 ± 6.18 and 27.4 ± 7.19 pmol · s^−1^ · mg^−1^, respectively (*P* > 0.05).

## Discussion

The current investigation constitutes the first systematic report on the influence of various methodological factors on the error in OXPHOS measurements performed on human skeletal muscle biopsies. We found relatively large variation (i.e., an SEM of 10.5 pmol · s^−1^ · mg^−1^ and CV of 15.2%) in repeated measurements involving the same biopsy. However, the routine procedure of assessing two bundles of fibers should reduce this SEM.

The variability in OXPHOS measurements involving the right and left thighs was similar to that between repeated measurements (SEM ~ 10 pmol · s^−1^ · mg^−1^), although the CV (~ 24%) was larger in this case due to the overall lower mean value. The SEM for the comparison between the two time‐points chosen was somewhat larger (15.2 pmol · s^−1^ · mg^−1^), although this difference was not statistically significant.

This similarity in methodological error associated with the different factors indicates that differences in sample preparation are the major cause of variability in measurements of OXPHOS on isolated bundles of muscle fibers. However, subject's circadian variability together with the intraindividual variations in, for example, nutrition and/or level of activity (van Moorsel et al. 2016) may also have contributed to the variation in the values obtained with the biopsies collected 27 ± 6 days apart. It is also possible that the mitochondrial pools in different bundles of muscle fibers are not identical, for example, due to variations in the fiber composition. However, if such heterogeneity were a major source of the methodological error observed, bundles composed of a larger number of fibers (i.e., heavier) should exhibit a smaller variation in OXPHOS than bundles with fewer fibers (i.e., lighter), which was not the case here (data not shown).

Another source of variation is of course the fact that all respiratory measurements are normalized to the wet weight of the fibers. To remove excess liquid, the permeabilized fibers were blotted on a filter paper for 5 sec without applying any external pressure. Longer blotting periods or pressing a filter paper over the fiber bundle would remove more liquid but also intracellular water and is not recommended. Alternative methods are recovering the fibers after the respiratory experiments and weighing them again or drying the fibers to assess fiber dry weight. However, a wet fiber weighing 2 mg would weigh only approximately 0.3–0.4 mg making the weighing procedure even more difficult. A third alternative would be to assess citrate synthase activity in the fiber bundles but again this has to be done after the respiratory experiments and it is unknown how the permeabilization procedure affects citrate synthase activity.

Our observation that OXPHOS measurements by two different technicians (Fig. 4) differ somewhat indicates the importance of having the same technician prepare, weigh, and permeabilize all of the samples for any given study. It is important to mention that the measurement error linked to the machine (i.e., high‐resolution respirometer) is not possible to measure because each observation is different from one another (i.e., different muscle bundles in each experiment even when obtained from the same muscle sample).

Importantly, the size of the muscle bundle had no influence on the OXPHOS values obtained. This indicates that with muscle bundles of ~ 0.5–4.5 mg in wet weight, diffusion of O_2_ does not limit OXPHOS. Furthermore, OXPHOS values remained stable for at least 5 h after the biopsy was collected (Fig. 6), indicating that BIOPS medium preserves mitochondrial function adequately during this period.

It is conceivable that additional factors may have contributed to the errors in measurement observed here, for example, the amount of local anesthesia applied (2–4 mL carbocaine), as well as the time that elapsed between injection of this anesthesia and collection of the biopsy (about 5–10 min), both of which were varied in response to the requirements of each individual subject. Long‐lasting exposure to local anesthetics have been reported to elevate levels of reactive oxygen species, altering muscle metabolism and thereby interfere with mitochondrial oxidative phosphorylation in preclinical models (Nouette‐Gaulain et al. 2012). Direct exposure of the fibers to carbocaine exerted no significant effect on mitochondrial respiration, but this does not definitely exclude that OXPHOS is impaired since the biopsy may already be influenced by the local anesthetic given to the subject before the biopsy collection.

It is difficult to determine the smallest meaningful change in OXPHOS in relation to performance, since this relationship may not be the same at baseline as after a training intervention that enhances both OXPHOS and performance (Granata et al. 2016). In controlled trials, the larger CV can be compensated for by performing duplicate measurements and/or recruiting more subjects. Furthermore, maximizing the measured O_2_ flux, using specific inhibitors that block contractile activity (i.e., N‐benzyltoluene sulfonamide and blebbistatin) (Perry et al. 2011) should reduce the CV due to the decreased ratio between the standard error of the measurement and mean of the measurements. However, from a practical standpoint in exercise physiology, 8–10 subjects are often recruited per group when comparing two population means; thus a difference of 15 pmol · s^−1^ · mg^−1^ between groups is necessary over the intervention effect on the basis of Equation (2) in the Material and Methods section.

On the basis of our present findings, we conclude that variations in OXPHOS measurements in permeabilized muscle fibers are primarily due to differences in sample preparation, indicating the importance of having one and the same technician preparing all samples for any given study. Furthermore, the OXPHOS values for muscle fibers originating from the left and right *vastus lateralis*, as well as for fibers collected from the same muscle at two time‐points 27 ± 6 days apart did not differ, both of which are important practical considerations. Moreover, there is no need to utilize muscle bundles of a standard weight, since with weights between approximately 0.5 and 4 mg, O_2_ diffusion was not limiting OXPHOS. Finally, muscle biopsies appear to be well preserved in ice‐cold BIOPS for at least 5 h after collection.

## Conflict of Interest

The authors have no conflicts of interest to declare.
